# Hepatocolic fistula in hepatocellular carcinoma treated with atezolizumab–bevacizumab: a case report on successful salvage hepatectomy

**DOI:** 10.1093/jscr/rjaf532

**Published:** 2025-07-17

**Authors:** Kota Ishida, Yuta Ushida, Takayuki Minami, Yoriko Yamashita, Masaya Inoue, Takehiro Kato, Ei Sekoguchi, Yasuhiro Kurumiya, Gen Sugawara

**Affiliations:** Department of Surgery, Toyota Kosei Hospital, 500-1 Josui-cho, Toyota, Aichi 470-0396, Japan; Department of Surgery, Toyota Kosei Hospital, 500-1 Josui-cho, Toyota, Aichi 470-0396, Japan; Department of Surgery, Toyota Kosei Hospital, 500-1 Josui-cho, Toyota, Aichi 470-0396, Japan; Department of Pathology, Toyota Kosei Hospital, 500-1 Josui-cho, Toyota, Aichi 470-0396, Japan; Department of Surgery, Toyota Kosei Hospital, 500-1 Josui-cho, Toyota, Aichi 470-0396, Japan; Department of Surgery, Toyota Kosei Hospital, 500-1 Josui-cho, Toyota, Aichi 470-0396, Japan; Department of Surgery, Toyota Kosei Hospital, 500-1 Josui-cho, Toyota, Aichi 470-0396, Japan; Department of Surgery, Toyota Kosei Hospital, 500-1 Josui-cho, Toyota, Aichi 470-0396, Japan; Department of Surgery, Toyota Kosei Hospital, 500-1 Josui-cho, Toyota, Aichi 470-0396, Japan

**Keywords:** hepatocolic fistula, atezolizumab, bevacizumab, hepatocellular carcinoma, salvage hepatectomy

## Abstract

Combined treatment with atezolizumab (ATZ) and bevacizumab (BV) is the first-line therapy for unresectable advanced hepatocellular carcinoma (HCC). We report a rare case of hepatocolic fistula during ATZ + BV therapy, successfully treated with salvage hepatectomy. A 72-year-old man with advanced HCC underwent treatment with ATZ + BV. After seven cycles, he presented with abdominal pain. Imaging revealed ascites and free air, leading to an emergency laparotomy, where panperitonitis was diagnosed. Despite lavage and drainage, postoperative fever persisted. Further imaging identified a liver abscess, requiring emergency ultrasound-guided percutaneous drainage. Contrast studies confirmed a hepatocolic fistula. The patient underwent ileostomy for abscess management, followed by extended posterior sectionectomy and right hemicolectomy on Day 25. He was discharged 37 days after final surgery without complications.

## Introduction

The combination therapy of atezolizumab (ATZ) with bevacizumab (BV) has emerged as a promising treatment for advanced hepatocellular carcinoma (HCC) [1, 2]. Adverse effects because of these immune checkpoint inhibitors can cause various immune-related adverse events, but hepatocolic fistula formation during ATZ + BV therapy has not been previously reported. We present a case of hepatocolic fistula caused by HCC that was successfully treated with extended posterior sectionectomy.

## Case report

**Figure 1:**
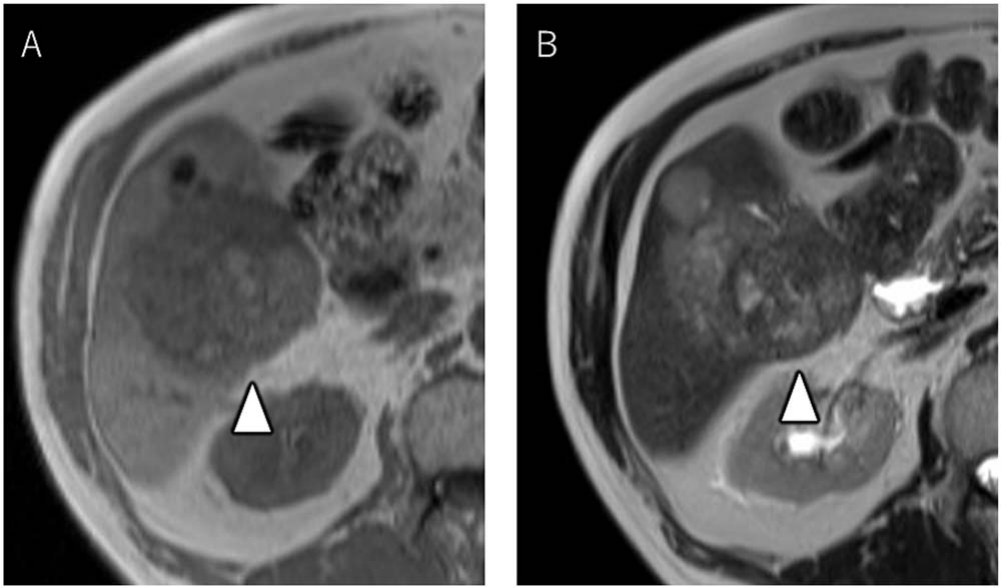
Magnetic resonance imaging showing multiple nodular lesions in liver S6/5 with low signal intensity on T1-weighted images (A), and mosaic-like high signal intensity on T2-weighted images (B).

**Figure 2:**
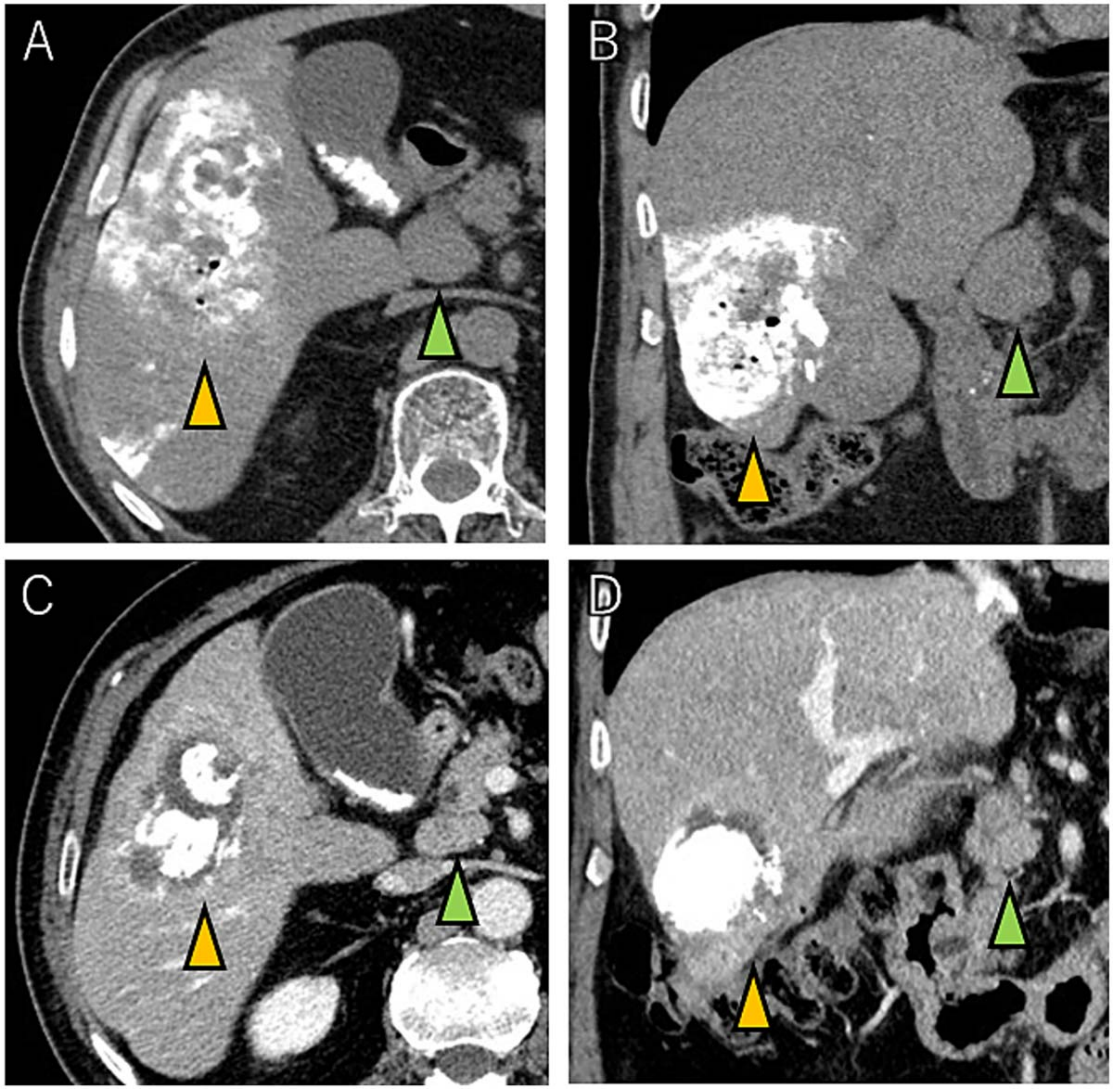
Tumor and enlarged lymph node in region 12b before ATZ + BV (A and B), and which got reduction after chemotherapy (C and D).

A 72-year-old man presented with abdominal pain. Computed tomography (CT) revealed a liver tumor in segment six/five, abutting the transverse colon without invading. Enlarged lymph nodes in regions 12b and 16b1 were noted, but there were no lung metastases or ascites. Magnetic resonance imaging revealed multiple nodular lesions in liver S6/5 with low signal intensity on T1-weighted images, and mosaic-like high signal intensity on T2-weighted images (Fig. 1). Based on these findings, the diagnosis of HCC was made, staged as cT2N1M1 Stage IVB. The patient underwent transarterial chemoembolization (TACE) followed by ATZ + BV therapy, resulting in tumor and lymph nodes reduction (Fig. 2).

**Figure 3:**
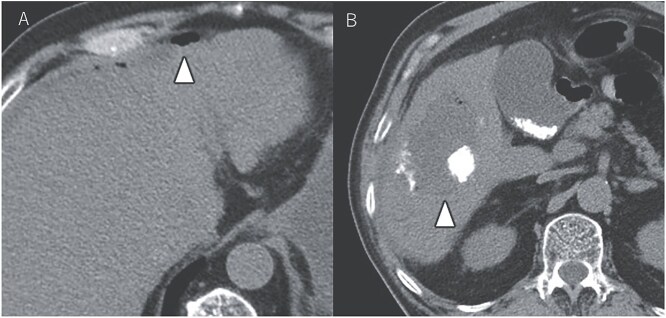
CT images showing tumor and enlarged lymph nodes in region 12b before ATZ + BV therapy (A and B). After chemotherapy, reduction of the tumor and lymph nodes is evident (C and D). Notably, no abscess formation was observed in the HCC or surrounding liver tissue, highlighting the direct communication between the tumor and the colon.

**Figure 4:**
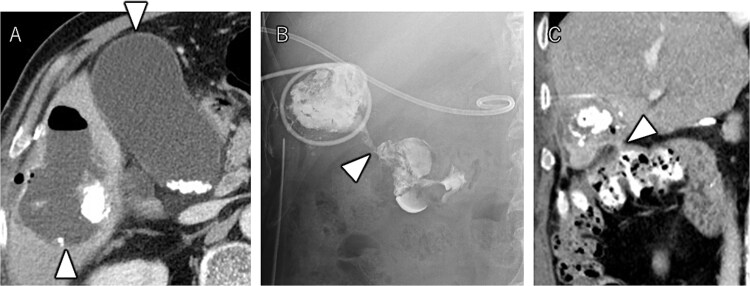
CT demonstrating a fluid collection with air inside the liver tumor, as well as enlargement and wall thickening of the gallbladder (A). Fistulography performing through the liver abscess drainage catheter revealed contrast filling in the colon (B). Subsequent CT scan showed that the liver tumor was adjacent to the catheter, with opacification of the colonic lumen (C).

**Figure 5:**
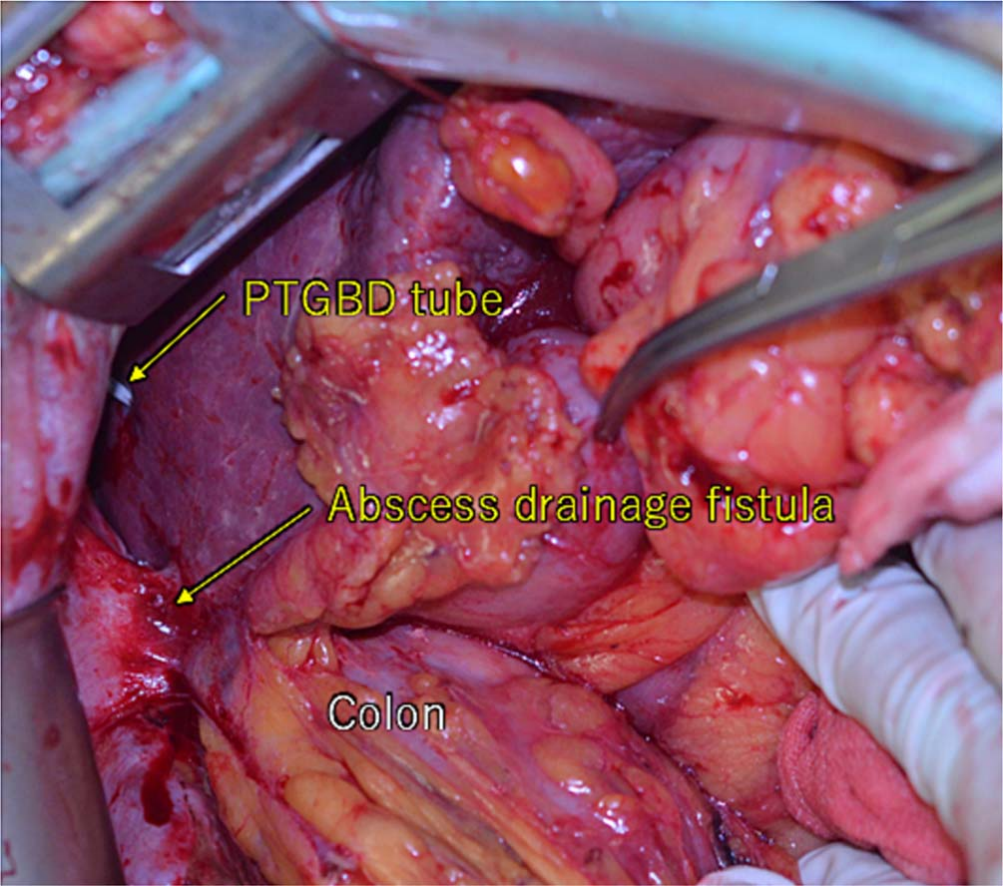
During the laparotomy, it was observed that the liver tumor, colon, and greater omentum were adhered together, forming a single mass. There were percutaneous transhepatic gallbladder drainage (PTGBD) tube and abscess drainage fistula. No intraperitoneal leakage of digestive fluids was observed.

**Figure 6:**
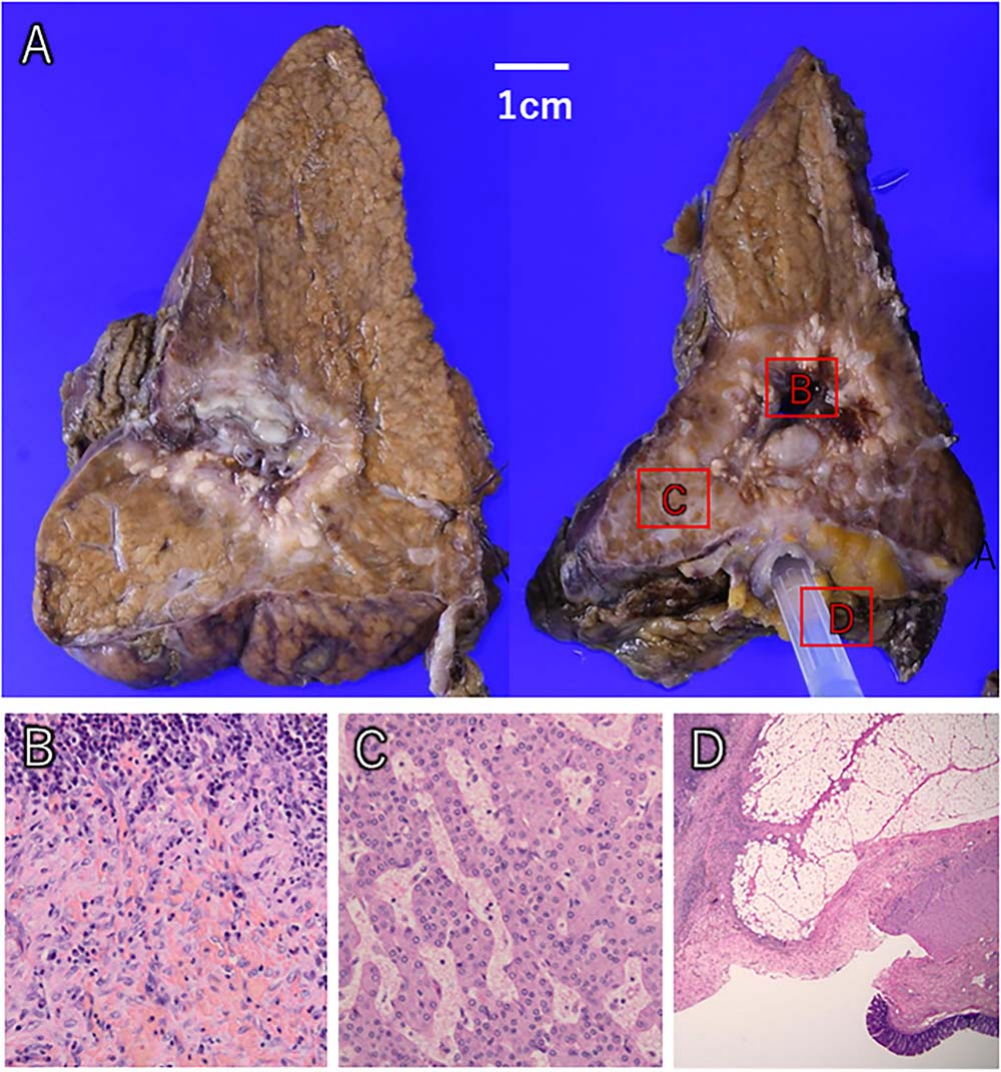
The liver tumor showed extensive macroscopic necrosis (A). Histologically, there was widespread necrosis with hemorrhage and inflammatory cell infiltration (B). Some areas exhibited proliferative cancer cells with disrupted trabecular structures, consistent with hepatocellular carcinoma (C). Colonic mucosa was identified within the fistula, confirming its formation between the liver tumor and colon (D).

**Figure 7:**
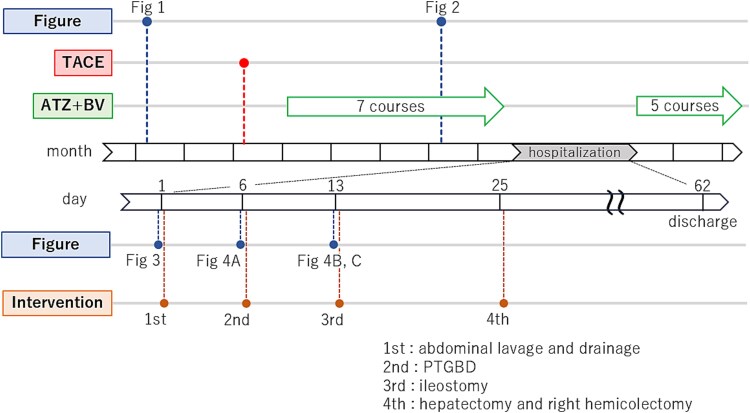
A time series diagram which shows the temporal relationship between figures and intervention, and, in particular, more detailed time series diagram of the hospitalization period.

After seven courses of treatment, he was transported to our emergency department because of abdominal pain. CT revealed ascites and free air on the liver surface as well as air within the liver tumor (Fig. 3). Peritonitis was diagnosed, and emergency laparotomy was performed. Strong adhesions were observed between the liver tumor, greater omentum, and the colon. Although small amounts of turbid ascites were found, no obvious perforations were observed in the stomach, duodenum, or small intestine. Abdominal lavage and drainage were performed. Postoperatively, persistent fever and elevated levels of inflammatory markers were noted. Subsequent CT revealed a fluid collection with air within the liver tumor and thickening of the gallbladder wall (Fig. 4A). The patient was diagnosed with a liver abscess secondary to HCC necrosis and cholecystitis, and percutaneous transhepatic drainage of the liver abscess and gallbladder was performed. Seven days after the drainage, fistulography through the liver abscess drainage catheter revealed colonic opacification (Fig. 4B). CT revealed the liver tumor adjacent to the catheter with opacification of the colonic lumen (Fig. 4C). Based on these findings, a diagnosis of hepatocolic fistula secondary to HCC was made. Considering the low likelihood of natural closure, loop ileostomy was performed 13 days after the initial surgery to improve the patient’s systemic condition, but fistulography showed no improvement. As a definitive treatment for the persistent hepatocolic fistula, salvage surgery of hepatectomy with concomitant colectomy was planned 25 days after the initial surgery. Intraoperatively, the liver tumor, colon, and greater omentum were adhered together as a single mass (Fig. 5). After partial diaphragm resection and hepatic lobe mobilization, extended posterior sectionectomy and right hemicolectomy achieved complete tumor removal without macroscopic residual disease. Macroscopically, the liver tumor showed extensive necrosis (Fig. 6A). Histologically, the majority of the tumor consisted of necrotic tissue with hemorrhage and inflammatory infiltration (Fig. 6B). Areas of disrupted trabecular structures with proliferative cancer cells were still visible, consistent with HCC (Fig. 6C). Colonic mucosa was identified within the fistula, unequivocally confirming the communication between the tumor and the colon (Fig. 6D). However, no significant abscess formation was observed in this area, reinforcing the hypothesis that the necrosis-induced weakening of the tumor boundary played a central role in the development of the fistula. No postoperative complications occurred and the patient was discharged on postoperative Day 37. Nine months postoperatively, the patient continued ATZ + BV therapy under the care of our department. The patient’s clinical course is summarized in the time series diagram (Fig. 7).

## Discussion

This is a case of advanced HCC with distant lymph node metastasis treated with TACE, followed by ATZ + BV therapy. To our knowledge, this represents the first reported case of a hepatocolic fistula developing during ATZ + BV therapy for HCC. This case highlights a unique interplay of tumor necrosis, inflammation, and localized anatomical changes contributing to the formation of a hepatocolic fistula without direct tumor infiltration or intermediate abscess formation.

Only eight cases of hepatic fistulae, including ours, have been reported in the past two decades. Among these, three cases were related to HCC, two to colonic foreign bodies (toothbrush and fishbone), two to infective hepatic abscesses, and one to residual intra-abdominal stones after cholecystectomy (Table 1) [3–9]. All three patients with HCC had a history of TACE. Case No. 5 underwent seven recent courses of TACE, while Case No. 3 underwent the last TACE 3 years before. Our case was 180 days prior to fistula formation, indicating a relatively weak association with TACE. In our case, ATZ + BV therapy likely led to tumor necrosis and subsequent hepatic abscess formation, resulting in inflammation extending to the adjacent colon and subsequent fistula formation. The interval between the onset of hepatic fistulae and therapeutic intervention ranged from 7 to 240 days. Hospital stays ranged from 12 to 63 days. In our case, poor infection control necessitated a staged therapeutic approach, including a highly invasive surgery, which resulted in a prolonged hospitalization until discharge.

**Table 1:** Published cases of hepatocolic fistula

Authors/year	Age (years)/sex	Etiology	Time to intervention (days)	Intervention	Hospital stays (days)
Kim/2007	31/M	Colonic foreign body (toothbrush)	7	Colectomy	N/A
Kim/2007	64/M	Colonic foreign body (fishbone)	14	Percutaneous catheter drainage	12
Kim/2011	76/M	RFA for HCC	N/A	Percutaneous catheter drainage	21
Stevens/2013	51/M	Liver abscess after laparoscopic cholecystectomy	240	Right hemicolectomy Resection of the hepatocolic fistula	17
Choi/2013	71/M	TACE for HCC	49	Sigmoidectomy with hepatectomy	20
Ishiwar/2015	45/M	Amoebic liver abscess	14	Percutaneous catheter drainage	21
Timbol/2017	58/M	Pyogenic liver abscess	120	Percutaneous catheter drainage	14
Our case/2024	72/M	ATZ + BV therapy for HCC	25	Percutaneous catheter drainage Hepatectomy and colectomy	63

This table summarizes reported cases of hepatocolic fistula, including patient demographics, etiology, time to intervention (days), intervention, hospital stays (days), and treatment. Our case involves a 72-year-old male on ATZ and BV for HCC, treated with percutaneous drainage, hepatectomy, and colectomy. M, male; F, female; HCC, hepatocellular carcinoma; HAC, hepatic adenosquamous carcinoma; RFA, radiofrequency ablation; TACE, transarterial chemoembolization; ATZ, atezolizumab; BV, bevacizumab.

BV has been associated with fistula formation, which can lead to gastrointestinal perforation [10]. Fistula formation and gastrointestinal perforation caused by ATZ due to immune-related adverse events (irAEs) are rare. However, irAEs can lead to enteritis, which may contribute to fistula formation or gastrointestinal perforation. To investigate the potential association between the drugs and the observed complication, we contacted Chugai Pharmaceutical Co., Ltd., the distributor of ATZ and BV in Japan. According to the company, there have been no previously reported cases of hepatocolic fistula related to these agents. While the possibility of enteritis induced by irAEs contributing to fistula formation cannot be ruled out, the precise mechanism remains unclear at this time. A literature search also revealed no similar reports. A somewhat related report described a case report of small intestine perforation during treatment with ATZ + BV therapy for HCC, where it was noted that ATZ-induced irAEs led to small intestine inflammation [11].

Surgical treatment of hepatocolic fistulas during chemotherapy is challenging because of factors including immunosuppression, systemic deterioration caused by inflammation, and the complexity of surgery. Due to the advanced cancer stage, poor overall condition of the patient, and the need for concomitant resection of other organs due to digestive tract invasion, many cases were deemed ineligible for surgery because of the highly invasive procedures required. In such situations, a staged surgical strategy may be adopted, beginning with infection control and temporary diversion, followed by definitive resection once the patient’s condition stabilizes. This approach may be referred to as “salvage surgery” when the primary intent is to control life-threatening complications such as persistent infection or fistula formation, rather than tumor eradication alone. In our case, where the infection was complicated by a hepatocolic fistula, salvage hepatectomy was considered under the following conditions: (1) good systemic status; (2) sufficient hepatic reserve; and (3) availability of facilities capable of safely performing hepatectomy. In our case, drainage and ileostomy was initially performed to control infection.

## Conclusion

This is a rare case of hepatocolic fistula that occurred during ATZ + BV therapy for advanced HCC, followed by salvage surgery for management. We hope that the accumulation of more surgical reports on hepatocolic fistulae will lead to the elucidation of appropriate surgical indications.
